# The essential requirement of an animal heme peroxidase protein during the wing maturation process in Drosophila

**DOI:** 10.1186/s12861-016-0143-8

**Published:** 2017-01-11

**Authors:** Dondra Bailey, Mohammed Abul Basar, Sanjay Nag, Nivedita Bondhu, Shaloei Teng, Atanu Duttaroy

**Affiliations:** 1 Biology Department, Howard University, 415 College Street, 20059 Washington, DC, NW USA; 2 Present address: Department of Cell and Developmental Biology, University of Pennsylvania Perelman School of Medicine, 19104 Philadelphia, PA USA

**Keywords:** Drosophila, Peroxidase, Wing development, Oxidase, Cysu

## Abstract

**Background:**

Thus far, a handful of genes have been shown to be related to the wing maturation process in insects. A novel heme peroxidase enzyme known as curly suppressor (Cysu)(formerly CG5873), have been characterized in this report because it is involved in wing morphogenesis. Using bioinformatics tools we found that Cysu is remarkably conserved in the genus Drosophila (>95%) as well as in invertebrates (>70%), although its vertebrate orthologs show poor homology. Time-lapse imaging and histochemical analyses have confirmed that the defective wing phenotype of Cysu is not a result of any underlying cellular alterations; instead, its wings fail to expand in mature adults.

**Results:**

The precise requirement of Cysu in wings was established by identifying a *bona fide* mutant of *Cysu* from the Bloomington Drosophila Stock Centre collection. Its requirement in the wing has also been shown by RNA knockdown of the gene. Subsequent transgenic rescue of the mutant wing phenotype with the wild-type gene confirmed the phenotype resulting from *Cysu* mutant. With appropriate GAL4 driver like engrailed-GAL4, the Cysu phenotype was compartmentalized, which raises a strong possibility that Cysu is not localized in the extracellular matrix (ECM); hence, Cysu is not engaged in bonding the dorsal and ventral cuticular layers. Finally, shortened lifespan of the *Cysu* mutant suggests it is functionally essential for other biological processes as well.

**Conclusion:**

*Cysu*, a peroxinectin-like gene, is required during the wing maturation process in Drosophila because as a heme peroxidase, Cysu is capable of utilizing H_2_O_2_, which plays an essential role in post-eclosion wing morphogenesis.

**Electronic supplementary material:**

The online version of this article (doi:10.1186/s12861-016-0143-8) contains supplementary material, which is available to authorized users.

## Background

In Drosophila, wings develop from fewer than 50 embryonic ectodermal cells located in the second and third thorasic parasegments [34], and a pair of wing imaginal discs are eventually established *via* metamorphosis from these cells [4, 5]. At the very early stages, *aristaless, vestigial*, *distal less, Escargot (Esg)* and *snail* gene products appear in specific order to establish an internal specification in the wing disc. Once established, multiple genes are expressed in a precise spatiotemporal order to determine the dorsal-ventral, proximal-distal and anterior-posterior axes of the wing imaginal disc. Developmental biologists have used many fine genetic tools to define the molecular events that take place in developing wing discs [25]. At this point, the cell-cell interactions in the wing imaginal disc are so precisely defined that it currently serves as a model to gain insight into the control of organ size in a three-dimensional tissue environment [3, 13].

During the pupal stage, a pair of wings evaginates from the wing imaginal discs. However, following its eclosion from the pupal case, wing morphogenesis is complete in adult flies. Approximately one hour after eclosion, through a rapid succession of events, blood or haemolymph is pushed into the wings. This process forces the wings to expand like a balloon, followed by the immediate withdrawal of the hemolymph inside the body cavity. Once the hemolymph is withdrawn, the dorsal and the ventral cuticular layers tightly bond together with the help of extracellular matrix (ECM) protein, thus forming the adult wings [17]. Afterwards, through a tanning process, a rigid wing structure is formed.

In comparison to the development of the wing imaginal discs, our understanding of the genetic process underlying pupal wing morphogenesis and adult wing expansion is quite limited. Gene expression profiles of early pupal wing structures have identified a few genes involved in hair and bristle morphogenesis and planar cell polarity [28]. Between eclosion and expansion, several major events occur in the wing, including the delamination of epithelial cells, the severing of cell contacts, the conversion of epithelial cells into mesenchymal cells, and the synthesis of ECM [17, 18]. Genetic manipulation studies showed that *Timp* (tissue inhibitor of metalloprotease), *Batone*, *pangolion*, *pygopus*, *shaggy/glycogen synthase,* and *αPS* integrin were related to the post-eclosion wing maturation process [16–19, 21]. The final events in wing morphogenesis are cuticle synthesis, sclerotization and melanization [1]. *Bursicon*, a neuropeptide hormone; *Dopadecarxylase* (Ddc); and the *pale* gene function are defined in the cuticular sclerotization and melanization process [7]. More recently, the action of *Duox* has been implicated in the final stages of the wing maturation process because RNAi knock down of *Duox* shows a pale and fragile wing phenotype that appears after wing expansion [2, 14, 15]. Biochemical analyses of *Duox* mutant wings revealed less tyrosine cross linkage, which ultimately affects the sclerotization and melanization of adult wings [2].

We report here that Cysu, a well-conserved animal heme peroxidase family of proteins, is also involved in post-eclosion wing expansion and the maturation process. An insertion mutation of *Cysu* leads to a recessive wing defect. While this study was in progress, Duox and the Cysu heme peroxidase were reported to potentially function collaboratively during the final stages of the wing maturation process [15]. Duox generates reactive oxygen species (ROS), including hydrogen peroxide, which is possibly utilized by the Cysu heme peroxidase during cuticular sclerotization. In relation to these findings, we also provide some evidence that a high ROS environment could be conducive to Cysu peroxidase induction.

## Results

### Cysu is a highly conserved heme peroxidase in invertebrates

**Fig. 1:**
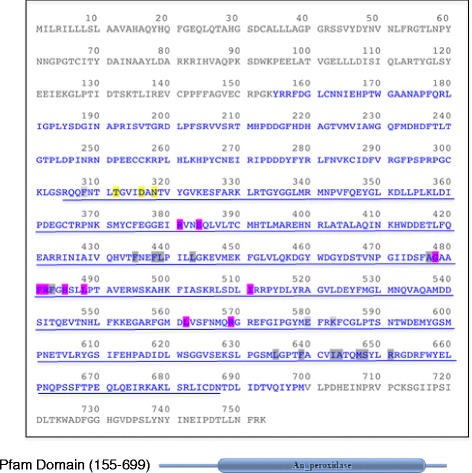
The sequence of the Drosophila Cysu protein. The animal heme peroxidase region is indicated in blue. Amino acids 155-699aa matches to Pfam entry. The site features a peroxinectin-like region that spans between 305-687 aa (*blue underline*). Calcium-binding sites (*yellow*); heme-binding sites (*pink*); putative substrate-binding site (*gray*) and three polypeptide-binding sites (homodimer interface at 308, 580, 583). The sequence was generated using MacVector

The Drosophila *Cysu* gene has a predicted open-reading frame of 753 amino acids with a predicted protein molecular mass of 85 kDa. The bulk of the Cysu protein consists of a heme peroxidase domain (Fig. 1) that is highly homologous to the animal-heme peroxidase superfamily. Embedded within the Cysu heme peroxidase domain is a specific sequence classified as the peroxinectin-like region (Fig. 1) [22]. In addition, the Cysu protein carries an integrin-binding domain (RGD) [33], several putative calcium-binding sites, substrate-binding sites, polypeptide-binding sites, and heme-binding sites (Fig. 1).

**Fig. 2:**
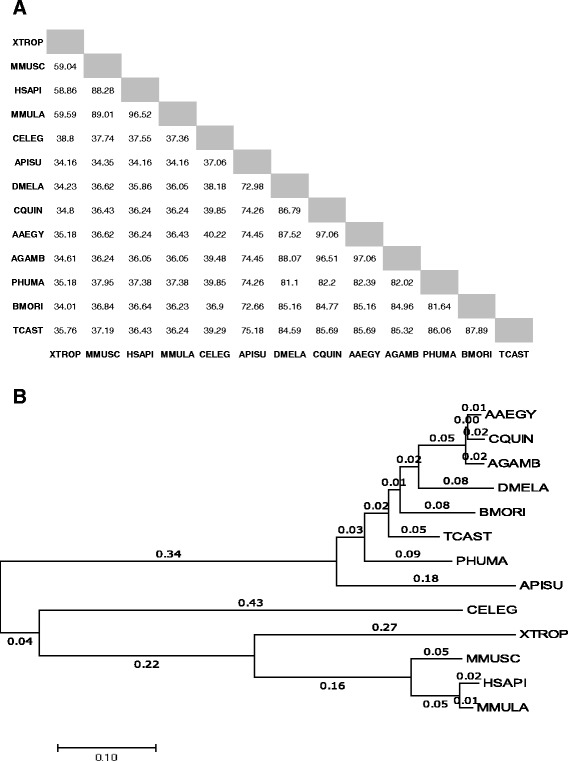
Evolutionary analysis of animal haem peroxidase domains between Drosophila Cysu with vertebrate and invertebrate proteins. (**a**) Percent Identity Matrix created with ClustalW 2 predicts the similarity in the cysu sequence to invertebrate and vertebrate proteins. Cysu is more than 72% identical to that of the invertebrates considered here except for *C. elegans*. The average identity of Cysu with vertebrates remained low at approximately 36%. (**b**) Phylogenetic Tree generated with MEGA7 indicates evolutionary relationships between Drosophila Cysu with vertebrate and invertebrate proteins. *Drosophila melanogaster* (DMELA), *Anopheles gambiae* (AGAMB), *Culex quinquefasciatus* (CQUIN)*, Acyrthosiphon pisum* (APISU), *Bombyx mori* (BMORI), *Tribolium castaneum* (TCAST)*, Pediculus humanus* (PHUMA), *Caenorhabditis elegans* (CELEG), *Homo sapiens* (HSAPI)*, Macaca mulatta* (MMULA), *Xenopus tropicalis* (XTROP), *Mus musculus* (MMUSC)

How conserved is Drosophila Cysu between different species of Drosophila? *Cysu* homologs are found in eleven diverse Drosophila species in addition to melanogaster with >95% identity between the different species (Additional file 1: Figure S1) which indicates that this protein may be engaged in essential biological function(s). To search for the Cysu homologous sequence among non-Drosophilid invertebrates as well as in vertebrates, a percent identity matrix (Fig. 2a) was computed among animal heme peroxidase domains of eight invertebrates and four vertebrate species using ClustalW.2 [20]. A Neighbor-Joining method was applied to a phylogenetic tree (Fig. 2b) for these domain sequences using MEGA7 [19, 29]. Overall, the Cysu protein is less identical (34% to 38% identity) to vertebrates. On the other hand, among invertebrate clusters, Cysu is remarkably conserved (>70% homology) with the non-Drosophilid invertebrates (Fig. 2a). One exception to this rule, however, is in *C. elegans,* where the percent identity score drops to 38%. This decreased homology could be related to the fact that *C. elegans* and other parasitic helminthes lack the heme biosynthetic pathway [27, 31].

### A Minos transposon insertion in the *Cysu* gene displays a recessive wing defect

**Fig. 3:**
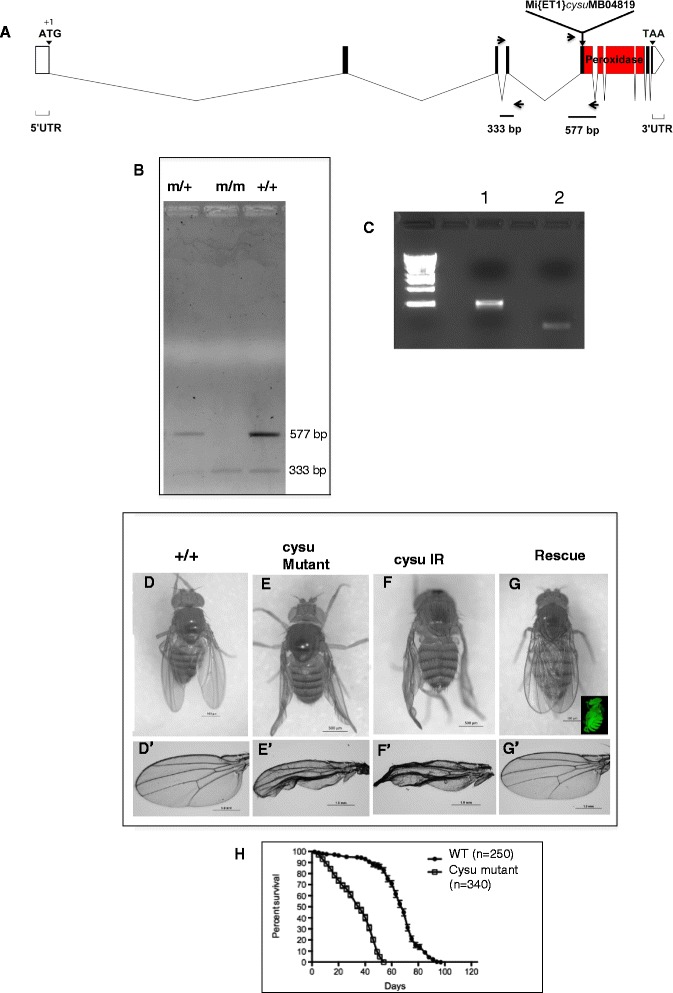
A single mariner insertion in the *Cysu* gene causes the collapsed wing defect. (**a**) A mariner insertion (MiET1) in the *Cysu* gene was available through Fly Base. Arrowheads indicate PCR primers encompassing the insertion point. (**b**) An expected 577-bp PCR band was amplified from wild-type (+/+) and insertion heterozygote (m/+) DNA. The same band is missing in the insertion homozygote (m/m). PCR amplification was performed with simultaneous amplification of an adjacent insertion-free region, which amplified a 333-bp DNA fragment in all genotypes. (**c**) A final confirmation on Minos insertion was obtained by amplifying a 540 bp fragment from inside the insertion (**F 5′** AAGAAAAACCGAAGTGCGCC 3**′ R**5**′** AGAGAGAACCGTCGCCAAAG 3′) and a 222 bp fragment by setting a primer inside the element and one outside in *Cysu* DNA (*Cysu:* F1 5′ TCGAAAAGTTGACGGCAGGA3′ Minos: R1 5′ TAGTGGTTGGGGCTCGTAGA 3′ (**d**) Wild type wing (**e**) A homozygous *Mi{ET1}* insertion in the *Cysu* sequence displays a severe wing defects in adults. (**f**) Activated *CysuRNAi* with a ubiquitous actin-GAL4 driver also shows the same collapsed wing phenotype seen in the insertion mutant. (**g**) Rescue of the collapsed wing phenotype with a *UAS-Cysu* ^*+*^ *GFP* transgene. Inset shows ubiquitous GFP expression with Actin-GAL4 driver. The mounted wings are shown from each genotype (D′-G′). (H) *Cysu* mutant adults have a significantly shorter life span compared to the wild type control

The *Cysu* gene is located on the third chromosome between the polytene intervals 90A6-90B1. An existing Minos based *Mi{ET1}* insertion *Mi{ET1}Cysu* ^*MB04819*^ was obtained from Fly Base (Fig. 3a). The location of this insertion was reconfirmed with primer sets designed across the insertion sequence (Fig. 3b) as well from inside the insertion (Fig. 3c), which suggests that the *Cysu* sequence is interrupted by the insertion which actually took place outside the peroxidase domain (Fig. 3a). To be precise, *Mi{ET1}Cysu* ^*MB04819*^ is inserted in exon 5 of *Cysu* gene at 17575 bp and 827 bp in Cysu cDNA starting from A(+1)TG (Fig. 3a).

Homozygous *y w, Mi{ET1}Cysu* ^*MB0419*^ flies are viable, with the adult animals displaying a severe wing defect (Fig. 3e). The manifestation of this wing tissue phenotype appears to be an unanticipated function in a peroxidase-like protein. Therefore, these findings raise immediate concern that a second site mutation or insertion may be responsible for the defective wing. We address this concern by knocking down the *Cysu* RNA expression*,* which showed a wing defect similar to the insertion mutant (Fig. 3f). Finally, complete rescue of the wing defect of the *Cysu* mutant was possible (Fig. 3g) by widely expressing a *Cysu* transgene (Fig. 3g inset). Together, we show that an altered function in *Cysu* is indeed responsible for the wing defect.

**Fig. 4:**
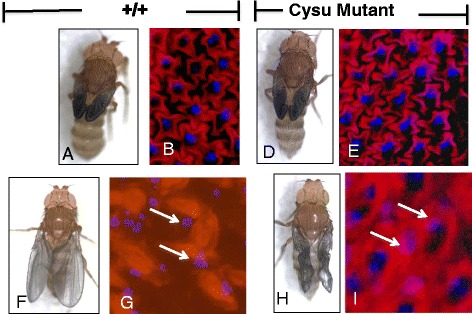
Analysis of epithelial cell morphology. The top row represents the morphology immediately after eclosion. **a** and **b** The wing epithelia appeared as star shaped cells immediately after eclosion as revealed by phalloidin staining. **c** and **d** The Cysu mutant wing maintains a similar star-shaped appearance with intact cell junctions. The bottom row represents wing expansion. **f** and **g** After the wings expand, star shaped epithelial cells lose contact with other cells, become circular and their nuclei become fragmented as revealed by DAPI staining. **h** and **i** Similar changes are evident in the *Cysu* mutant wing as well

To further define the Cysu phenotype, we examined the cell-cell adherens junctions in the *Cysu* mutant wing by Phalloidin staining. The adherens junctions appear intact in this mutant similar to wild type (Fig. 4). Similarly, with respect to the dorsal and ventral wing layers, they are completely reapposed in the mutant wing such that no gaps or fluid-filled blisters appeared in the mutant wing (Figs. 3e, e’ and f, f’). These two observations relate to the fact that Cysu is not involved in the function of adherens junctions nor does it affect the ECM proteins.

In addition to its requirement in the wing, the function of Cysu could be essential for other physiological processes as well. Therefore, we set out to measure the lifespan of the *Cysu* mutant as attaining a normal lifespan with no indication of premature death is considered to be a measure of good health [32]. *Mi{ET1}Cysu* ^*MB0419*^ flies were made isogenic with the *w* ^*1118*^ *; +/+* control strain through repeated backcrossing (~8-10 generations). After the backcross, the median lifespan of *Cysu* mutant flies was determined to be 34 days, which revealed that, in comparison to the control strain, the *Cysu* mutant flies exhibited a 49% reduction in median lifespan (Fig. 3h). Incidentally, ubiquitous knockdown of *Cysu* with *Act5C-GAL4 > CysuIR* or *Tub-GAL4 > CysuIR* resulted in extremely weak flies that survived up to 3-4 days at most (data not shown), while knocking down *Cysu* with *Da-GAL4 > CysuIR* resulted in flies that had the same lifespan as the mutant. The observed discrepancy in survival lengths between the *Cysu* mutant and Cysu knockdowns with certain ubiquitous drivers may have resulted from either the driver strength or its temporal expression or an off target RNAi effect in the rest of the body. But the wing defect remains same in all cases.

### *Cysu* mutant appears from faulty wing expansion process

By knocking down *Cysu RNA* expression from the early wing disc with the help of several early GAL4 drivers such as *Aristaless-GAL4, DPP-GAL4, distaless-GAL4, hedgehog-GAL4, vestigial-GAL4, wingless-GAL4 and dorsal wing-GAL4*, we found no effect no effect as the wings formed normally (Additional file 1: Figure S3). These findings indicate that Cysu is not functionally necessary during wing disc development.

**Fig. 5:**
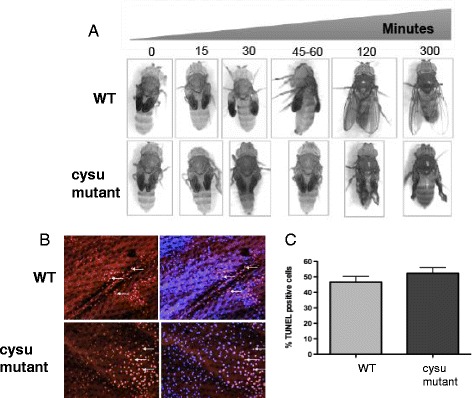
Time-lapse imaging of the wing expansion process. **a** The time from eclosion until the beginning of expansion (45-60 minutes total) in both wild type and Cysu mutant wings. The same fly was photographed at the noted intervals. At around the time when wing expansion begins, the *Cysu* mutant wings fail to properly expand, resulting in a collapsed appearance. **b** Cell death occurs naturally in the wings following expansion. Red dots correspond to TUNEL-positive nuclei (arrows). Cell death occurs in the Cysu mutant wings as well. **c** The percentage of TUNEL-positive cells shows no significant increase in the mutant wings (*n* = 3) on the two-tailed t-test 95% confidence interval; *P* = 0.34

A time-lapse video series was used to pinpoint exactly when the mutant wings began to collapse during adult life. Immediately after eclosion until prior to expansion, the *Cysu* mutant wings appeared similar to the wild-type wings (Fig. 5a), which also eliminates the requirement of Cysu in the development of the wings during the late pupal stage or in early adulthood. The wings begin to expand at approximately 60 minutes post-eclostion (see Additional file 2: Movie S1 and Additional file 3: Movie S2). Precisely around this time, the *Cysu* mutant wing fails to expand properly as shown in Fig. 5. Video image documents very clearly that the mutant wing begins to expand but the wing is crumpling both from dorsal and ventral sides. Additionally, we also noticed that the Cysu wings first appeared as short and crumpled but the phenotype continue to worsen with time and eventually it took a tendril like appearance (Additional file 1: Figure S2). Therefore, we hypothesize that Cysu function may be required during wing expansion and the subsequent cuticular sclerotization process (Fig. 5a) although any earlier requirement can not be ruled out at this point. Finally, we investigate whether this sudden wing collapse is related to a burst in epithelial cell death. No excess cell death was observed in the *Cysu* mutant wings as revealed by TUNEL staining of the mutant wings during this time interval (Figs. 5b and c).

**Fig. 6:**
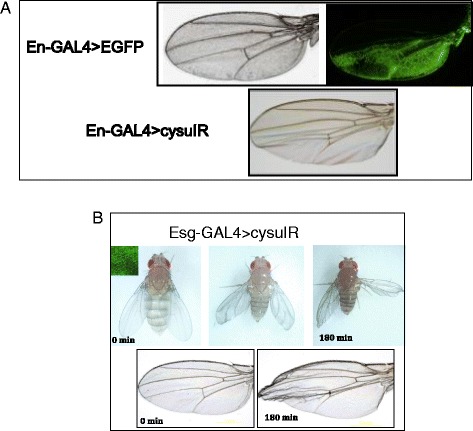
The cell-autonomous nature of Cysu. **a** Engrailed-GAL4 > EGFP expression is limited to the posterior compartment of the adult wing. Activation of *CysuIR* with the Engrailed GAL4 driver causes the wing morphology to change only in the posterior compartment. The posterior part of the wing appears extremely kind of flimsy. **b** A time-lapse video of an *Esg-GAL4 > CysuIR* fly shows that the wings form and expand completely normally in these flies. However, after a few hours, the wings begin to deteriorate. After approximately 3 hours, a complete expansion the wings show a collapsed appearance


Additional file 2: Wing expansion video for wild type. (MOV 1515 kb)



Additional file 3: Wing expansion video for Cysu mutant. (MOV 1869 kb)


### Inactivation of Cysu in the posterior compartment limits the wing defect mostly to that part of the wing

**Fig. 7:**
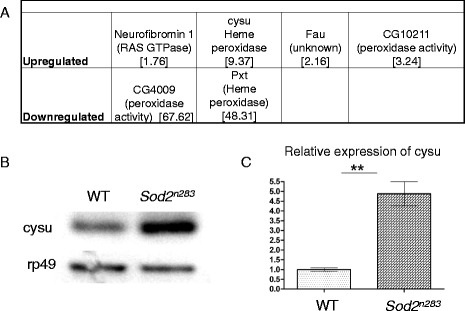
Cysu expression in a high ROS environment. **a** Gene array profiles of a *Sod2* null mutation [10], which is known to produce high amounts of mitochondrial ROS [24]. While Cysu is highly upregulated in this mutant, but not all peroxidases are and some are significantly down regulated in the same environment. **b** An RT-PCR analysis confirmed that Cysu transcripts are upregulated approximately five fold in this highly prooxidative environment in the *SOD2* mutant

Engrailed is expressed in the early wing disc at the posterior compartment. Its expression continues to persist in the adult wing and remain restricted mostly to the posterior compartment (Fig. 7a) [2]. We used the *engrailed GAL4* driver to knockdown Cysu RNA expression from the posterior compartment of the wing. In *EngGAL4 > CysuIR* flies, only the posterior compartment of the adult wing appeared flimsy and not so well formed (Fig. 6a) whereas the anterior compartment appeared normal (Fig. 6a). This finding is significant and quite relevant becasue proteins involved in ECM formation are capable of diffusing out to long distance. For example *Timp* (tissue inhibitor of metalloprotease), forms *Timp* ^*-/-*^ cell clones in the wing but being a matrix protein Timp can diffuse out to the adjacent cells [17] hence no effect of Timp loss of function was evident in the clones. In this case the effect of Cysu knockdown in the posterior compartment remain in the same compartment. Although we can’t address whether Cysu is intra- or extra-cellular but this data suggests it could be extracellular, but not diffusible.

### Inactivation of Cysu can affect wing morphology after expansion

*Escargo*t (*Esg*) expression is required very early for the specification of wing primordial cells because *Esg* helps epithelial cells attain an ectodermal cell fate [12]. Expression of *CysuIR* with *Escargot-GAL4* resulted in adults whose wings expanded normally and whose wings formed with no distortion (Fig. 6b). However, a time-lapse video showed that 3 hours after the expansion of the wings was complete, the wings began to collapse in *Escargot-GAL4 > Cysu IR* flies, and ultimately, a collapsed wing phenotype resulted as in the *Cysu* mutant (Fig. 6b). These data confirmed that Cysu function is absolutely required for the maintenance of adult wing structure, although it was not clear why the effect appeared so much later. In this context, Cysu mimics the effect of Duox on the wing, which appears 24 hours post-expansion and continues to get worse [2].

### A high ROS environment may be conducive to the induction of Cysu

Mitochondrial superoxide dismutase (MnSOD or SOD2) is the principal ROS metabolizing enzyme in mitochondria [24]. Drosophila mutant for *SOD2* gene, *Sod2* ^*n283*^, suffers from mitochondrial dysfunction in the absence of SOD2 and generates high levels of ROS [10, 23]. A gene array analysis performed with *Sod2* ^*n283*^ flies and a search for oxidative stress response genes in this prooxidative environment showed that Cysu mRNA is upregulated approximately 9.37-fold higher in *Sod2* ^*n283*^ than in the control (Fig. 7a). Notably, this upregulation is not universal to all heme peroxidases because pxt (peroxynectin like) and CG4009 were significantly downregulated in this high ROS environment (Fig. 7a). The validation of microarray data with RT-PCR confirmed that *Cysu* transcripts are indeed upregulated approximately 4.5-fold in *Sod2* ^*n283*^ flies (Fig. 7b and c). Is Cysu peroxidase induced as a complementary response to the loss of SOD2 function? We examined this possibility by overexpressing Cysu in the *Sod2* ^*n283*^ mutant background, but failed to rescue the neonatal lethality of the loss of function of *Sod2*. However, the fact that Cysu may be a ROS sensitive gene is informative in itself.

## Discussion

A detailed characterization of the Cysu heme peroxidase protein with regards to its role in the insect wing maturation process is reported here. It is quite clear from our analysis that Cysu heme peroxidase is particularly well conserved among invertebrates. Second, the Cysu peroxidase carries a peroxinectin signature with its integrin-binding motif, Arg-Gly-Asp (RGD), which is similar to the peroxinectins previously described in the crayfish and in the black tiger shrimp *Penaeus monodon* [33]. No vertebrate peroxinectin has been reported so far. Shrimp peroxinectin is highly similar to *Drosophila* peroxinectin-related proteins, except that shrimp protein actually carries two integrin-binding motifs, RGD and Lys-Gly-Asp (KGD) [33]. Integrins are primarily involved in cell adhesion. Therefore, loss of *PS1 integrin* function causes prominent fluid-filled blisters in the Drosophila wings because the dorsal and ventral wing layers fail to reappose and form stable junctions. This finding led us to ask whether the single integrin-binding motif in Cysu can be engaged in adhesion function in Drosophila. There was considerable doubt, as neither the *Cysu* mutant nor RNAi ablation of this gene showed a blistery wing phenotype, and we failed to locate the separation between the dorsal and ventral cuticular layers anywhere in the collapsed wings despite our best effort. Second, the adherens junctions or the cell-to-cell contact points appear completely intact in the *Cysu* mutant wings. Finally, by nature, adherens proteins need to stay in the ECM so that they can diffuse over long distances [17]. Because Cysu can be compartmentalized, therefore its localization in the ECM can not be justified. Therefore, Cysu is not engaged in adhesion function between the dorsal and ventral layers.

**Fig. 8:**
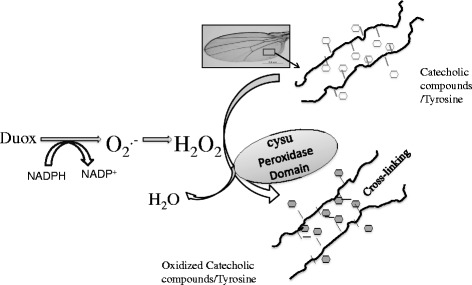
The current understanding of the action of the Cysu heme peroxidase in wing morphogenesis. The peroxidase domain of Cysu utilizes Duox, which generates H_2_O_2_ to oxidize catecholic compounds and tyrosines that are present in the protein chains of the cuticle [1, 2, 11]. The oxidation of catecholic compounds and tyrosines helps in cross-linking the protein chains, a process that hardens the wing structure

If Cysu is presumably not engaged in adhesion function and if our analysis also ruled out its requirement during wing disc development how is wing expansion affected in the Cysu mutant? It was recently proposed that both Cysu heme peroxidase and Duox may be required in the insect wing maturation process [15]. Duox is able to generate superoxide (O_2_^.-^) radicals or hydrogen peroxide (H_2_O_2_) by transferring electrons from NADPH to oxygen through FAD in mammalian cells [8, 9]. However, Drosophila Duox may have lost its ability to utilize H_2_O_2_ because it lacks several crucial amino acids [15], although in vitro assays with Duox refute this claim [14]. From biochemical studies on insect cuticular sclerotization, it is evident that various catecholic compounds and tyrosines act as precursors for cross-linking the protein, which helps in the stabilization of the wings following eclosion [1]. The lack of Duox activity was reported to lead to reduced levels of catecholic compounds and dityrosine residues in the wings, which likely explains the fragile wings [2] and the Curly (Cy) wing phenotype of the Duox mutant [15]. As for Cysu, heme peroxidases are capable of generating H_2_O_2_ and thus help in oxidizing catecholic compounds and tyrosines for crosslinking proteins [1]. Now we have shown that *Cysu* mutant has a late-appearing wing phenotype, just like Duox. We therefore argue that Cysu heme peroxidase utilizes Duox-generated H_2_O_2_ to oxidize catecholic compounds (Fig. 8). In the Cysu mutant wing such utilization of H_2_O_2_ doesn’t happen which affects oxidation of catecholic compounds and tyrosine crosslinking in the mature wing. Many questions remain, however, about the possible interaction between Duox and Cysu peroxidase in the wing, particularly when knockdown of Cysu in the Duox mutant with the Cy wing phenotype suppresses the Cy phenotype, making the Cy wings appear almost normal [15]. Fortunately, *bona fide* mutants of *Duox* and *Cysu* are now available, which will enable us to perform more interaction studies and biochemical analyses of the double mutant. The availablity of these two mutants will also eliminate the involvement of the GAL4-dependent system, which very much depends on the temporal requirement of the driver and driver strength. Finally, a Cysu-Duox partnership could be essential for other biological function(s) as well, such as pathogen resistance. These findings can thus explain the shortened lifespan of the *Cysu* mutant.

## Methods

### Drosophila fly stocks and husbandry

All fly stocks were maintained at 24 ± 1 °C on standard agar-yeast-sucrose medium under constant humidity and a 12 h:12 h light-dark cycle. A detailed list of the genotypes of the fly stocks used in this study and their sources are listed below:

(A) *w; Mi{ET1}Cysu* ^*MB04819*^ */TM6C, Sb* ^*1*^ (Fybase ID 0024824) was obtained from the Bloomington Drosophila Stock Center (BDSC). (B) *w; P{GD6190}v14374* is the *Cysu* RNAi line (Stock Number v14374) that was obtained from the Vienna Drosophila RNAi Center (VDRC, Austria). (C) The rest of the fly stocks, including all of the GAL4 lines that we used, came from BDSC. (D) The generation of the UAS-Cysu-GFP fusion construct: Gateway Technology was used to make transgenic insertion lines. Transgenic flies expressing the GFP–Cysu fusion protein were generated by cloning the *Cysu* cDNA into *pPGW (DGRC Stock number 1077)* vectors.

### DAPI, phalloidin and TUNEL staining

For DAPI and phalloidin staining, the wings were fixed in 4% paraformaldehyde for 1-2 hours, washed with PBST (2x) for 10-15 min and stained with phalloidin (1 μg/mL). After the washing steps, the samples were stained with DAPI (250X Sigma) and mounted with the Vectashield Mounting Medium (Vector Laboratories Cat. No H-100).

For TUNEL staining, the in situ Cell Death Detection Kit and TMR red were used (Roche Diagnostics). Whole flies were fixed in 4% paraformaldehyde in PBS/heptane for 2 hours and permeabilized in phosphate buffer saline with Triton X (PBX) for 30 min. The fixed samples were removed and subjected to proteinase K (200 ug/mL) digestion in 10 mM Tris-HCl pH 7.4-8. Subsequently, samples were incubated for at least 2 hours in the TUNEL reaction mixture containing TdT and TMR-dUTP according to the manufacturer’s instructions. TUNEL-positive nuclei were visualized on mounted wings using fluorescence microscopy. Statistical analyses were performed using the GraphPad Prism version 5.0b software (LaJolla, CA). Statistical comparisons were determined by Student’s *t-*tests. Values of *p* < 0.05 were considered statistically significant.

### Microscopy and Imaging

Specimens (including fly wings) were analyzed using transmitted light and incident-light fluorescence. The images of wings were mounted on glass slides and photographed using a bright field Axioplan 2 Zeiss microscope equipped with an Olympus camera. Whole flies and, in some cases, movies were imaged with the AxioCam MRc5 attached to a Zeiss stereoscopic microscope. Further processing was conducted with the Zeiss Axiovision imaging software. Confocal microscopy was performed on a Nikon spinning disc (Andor Zyla sCMOS) using 40x or 60x oil immersion objectives. In some cases, overlays or z-stack projections (z-intervals of typically 0.1 - 0.5 μm) were taken. Images were further processed with the Nikon NIS Elements Imaging Software (Version 4.20.01) (Advanced Research).

### Lifespan analysis

Life span studies were conducted on standard Drosophila medium with at least three replicate experiments at 24 ± 1 °C [26]. Female flies were collected immediately after eclosion and mated for approximately 3 days. Females were distributed at a density of 10-15 flies per vial and changed on fresh media every two days. The number of dead animals was determined daily. Log-rank and Wilcoxon tests were used for statistical analysis using the program GraphPad Prism version 5.0b.

### Bioinformatics analyses

The Cysu (FlyBase ID: FBgn0038511) protein sequence was analyzed using NCBI Protein Blast and PROSITE to identify the protein domains and family and functional sites. Additionally, the functional sites, patterns and profiles associated with the superfamily (peroxidase 3) were identified using the PROSITE Database and ScanProsite [30].

### Multiple Sequence Alignments

*Drosophila* Cysu orthologs were obtained from FlyBase (OrthoDB), and a multiple sequence alignment was generated to determine the percent similarity between the orthologs. All *Drosophila* orthologs of Cysu selected with Gene ID/UniProt. Sequences were aligned using Clustal Omega (EMBL-EBI) to determine the protein matrix and percent similarity. The Clustal Omega program is available at http://www.ebi.ac.uk/Tools/msa/clustalo/. To verify the accuracy, two additional alignment applications were employed: 1) The PRofile ALIgNEment (PRALINE), a fully customizable application that generates an extended multiple alignment with (The E-value threshold 0.01) and 2) MutiAlin version 5.4.1 [6]. For ease and optimal visual comparisons, MultiAlin alignment profile consensus sequences are shown in our study under the following alignment parameters: Symbol comparison table using blosum65, Gap weight of 12, and Gap length weight of 2. The consensus symbols are indicated in the last row of each alignment.

## Additional file

Additional file 1: Figure S1. Alignment of Cysu with Drosophila orthologs**.** The highly conserved peroxidase region indicated by the consensus line appears in red, uppercase letters; a weak conservation is indicated in lowercase blue. Drosophila species are highly conserved (~95%) among the following: *D. grimshawi* (DGRIM), *D. mojavensis* (DMOJA), *D. virilis* (DVIRI), *D. willistoni* (DWILL), *D. pseudoobscura* (DPSEU), *D. ananassae* (DANAN), *D. erecta* (DEREC), *D. yakuba* (DYAKU), *D. sechellia* (DSECH), and *D. simulans* (DSIMU)*, D. persimilis* (DPERS). The green line indicates the peroxinectin region. **Figure S2.** Severity of Cysu phenotype changes with time. Flies were collected (Day 1) photographed at different time interval until Day 2. In all cases the phenotype gets more and more severe indicating that the entire wing was affected. **Figure S3.** Knockdown of Cysu expression in each essential compartment of the wing disc cause no effect on wing development because adult wings develop normally. Except for Actin-GAL > CysuIR where ubiquitous suppression results in collapsed wing effect. (PPT 17161 kb)

## Abbreviations

DAPI: 4′,6-diamidino-2-phenylindole
PBST: Phosphate Buffered Saline with TWEEN-20
ROS: Reactive Oxygen Species
SOD: Super Oxide Dismutase
TUNEL: Terminal deoxynucleotidyl transferase (TdT) dUTP Nick-End Labeling (TUNEL)
