# Supplementary material for: The essential requirement of an animal heme peroxidase protein during the wing maturation process in Drosophila
Source: BMC Dev Biol. 2017 Jan 11;17:1. doi: 10.1186/s12861-016-0143-8 (PMC5225594; doi:10.1186/s12861-016-0143-8)
Supplement: Additional file 1: Figure S1. — Alignment of Cysu with Drosophila orthologs. The highly conserved peroxidase region indicated by the consensus line appears in red, uppercase letters; a weak conservation is indicated in lowercase blue. Drosophila species are highly conserved (~95%) among the following: D. grimshawi (DGRIM), D. mojavensis (DMOJA), D. virilis (DVIRI), D. willistoni (DWILL), D. pseudoobscura (DPSEU), D. ananassae (DANAN), D. erecta (DEREC), D. yakuba (DYAKU), D. sechellia (DSECH), and D. simulans (DSIMU), D. persimilis (DPERS). The green line indicates the peroxinectin region. Figure S2. Severity of Cysu phenotype changes with time. Flies were collected (Day 1) photographed at different time interval until Day 2. In all cases the phenotype gets more and more severe indicating that the entire wing was affected. Figure S3. Knockdown of Cysu expression in each essential compartment of the wing disc cause no effect on wing development because adult wings develop normally. Except for Actin-GAL > CysuIR where ubiquitous suppression results in collapsed wing effect. (PPT 17161 kb) [file 12861_2016_143_MOESM1_ESM.ppt]

## Slide 1
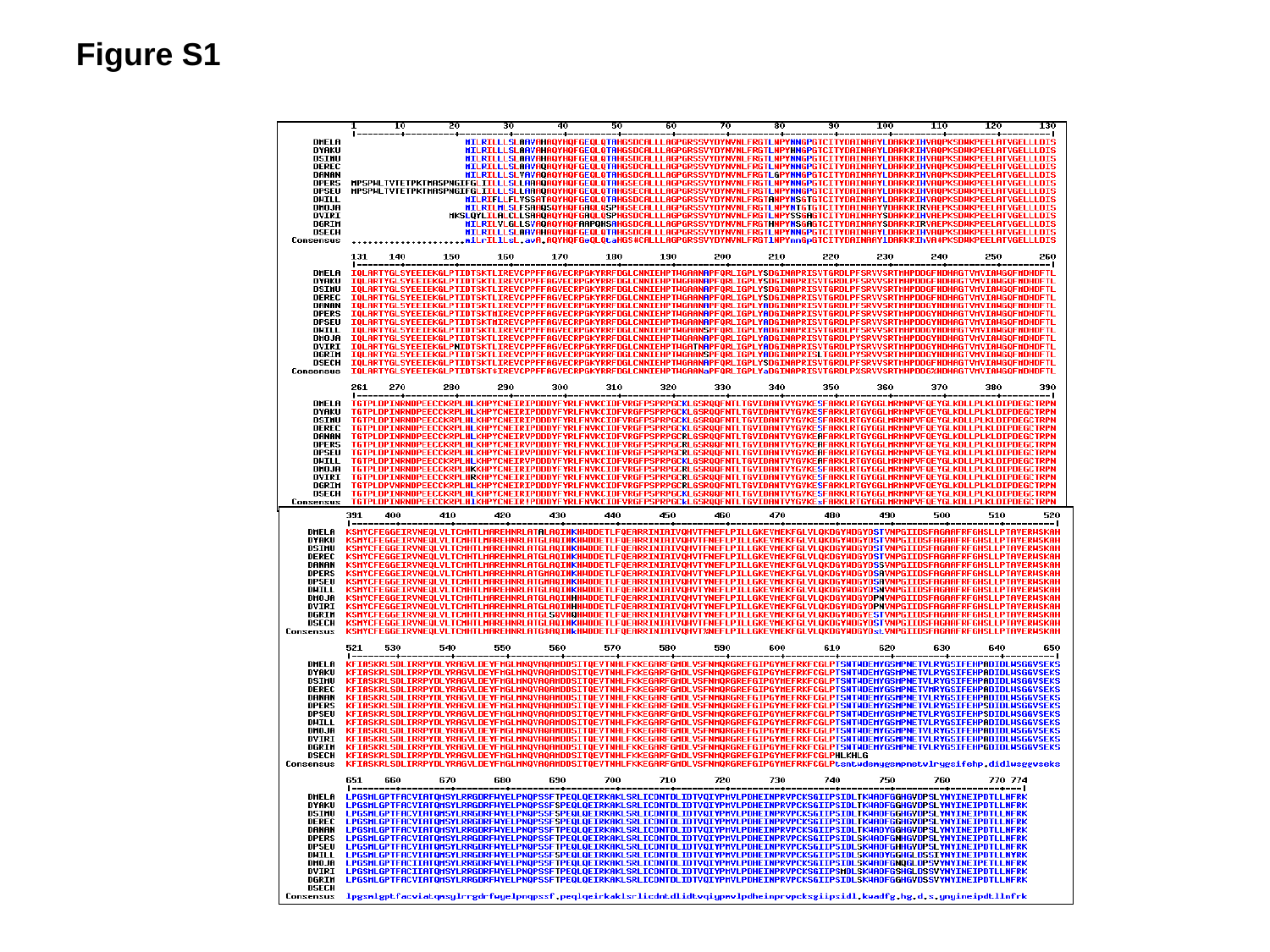

Figure S1

## Slide 2
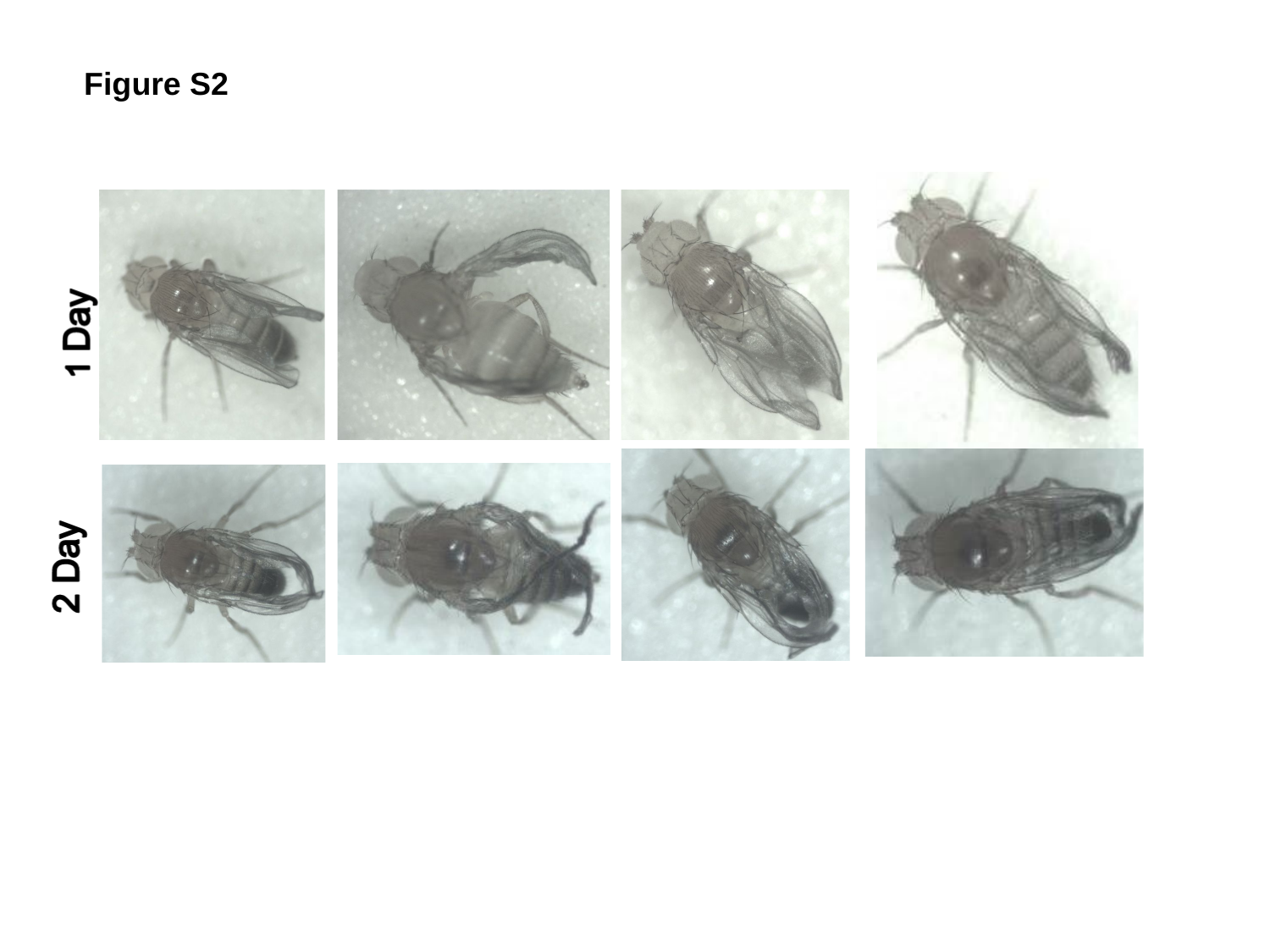

Figure S2

## Slide 3
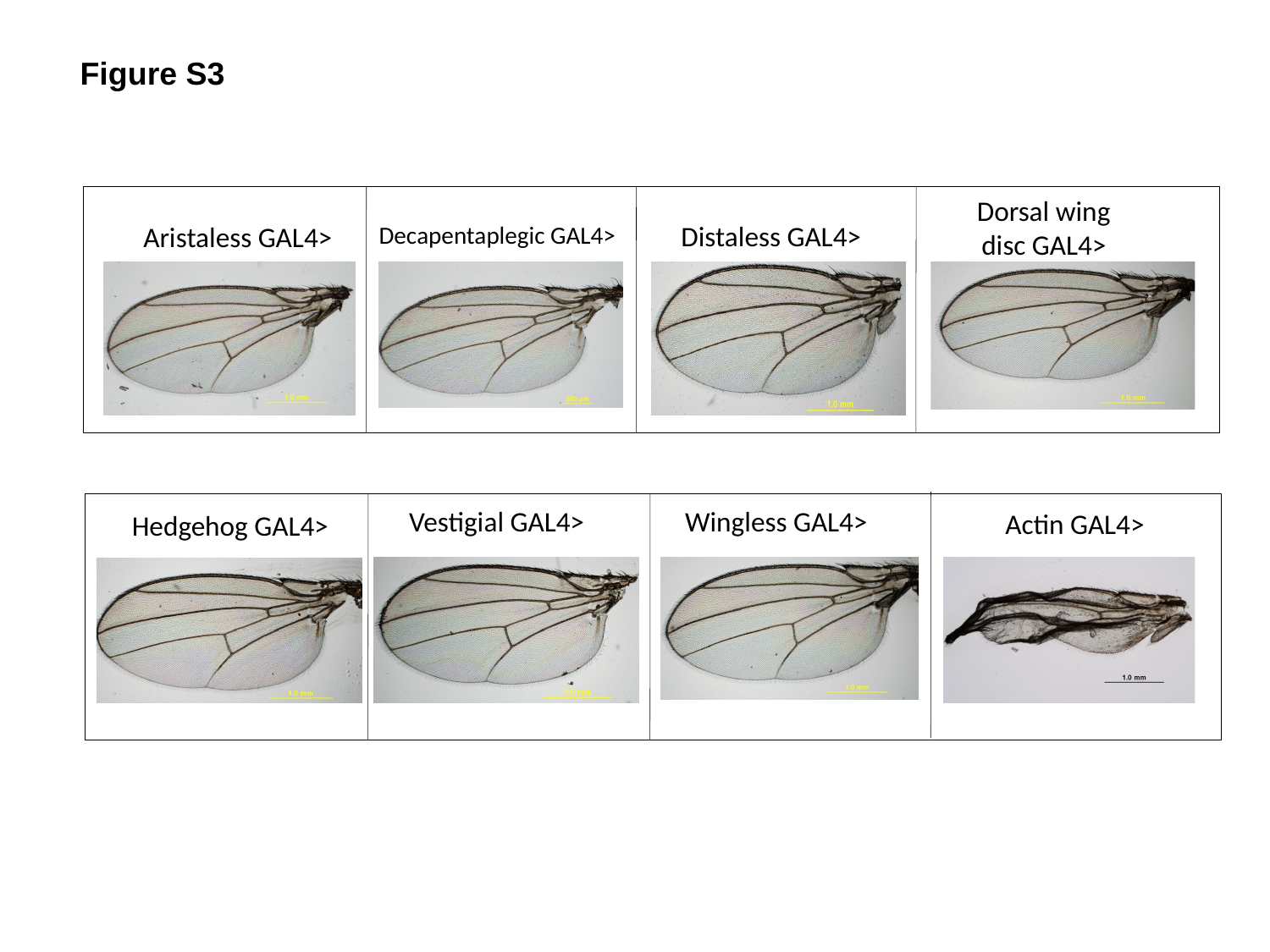

Figure S3
Dorsal wing
disc GAL4>
Distaless GAL4>
Aristaless GAL4>
Decapentaplegic GAL4>
Vestigial GAL4>
Wingless GAL4>
Actin GAL4>
Hedgehog GAL4>
